# The costs and benefits of diagnosis of ADHD: commentary on Holden et al.

**DOI:** 10.1186/1753-2000-8-7

**Published:** 2014-03-01

**Authors:** Ginny Russell, Tamsin Ford

**Affiliations:** 1 Institute of Health Research, University of Exeter Medical School, Veysey Building, Salmon Pool Lane, Exeter, UK

**Keywords:** Attention deficit hyperactivity disorder, ADHD, Prevalence, Healthcare costs

## Abstract

In this journal, Holden, Jenkins-Jones, Poole, Morgan, Coghill and Currie , CAPMH 7:34, 2013, report on the prevalence and financial costs of treating people with attention deficit hyperactivity disorder (ADHD) in the UK over the last ten years. We commend the authors on their thorough cost analysis, and discuss differences in prevalence estimates of diagnosed ADHD, that is the proportion of the child population with an ADHD diagnosis, which varies dramatically between studies. We also discuss the reasons for this. Regional variation in application of diagnostic criteria and clinical subjectivity are likely partial explanations.

## Background

Holden, Jenkins-Jones, Poole, Morgan, Coghill and Currie [1] perform a thorough analysis of the costs of treating ADHD, and estimate that the added overall cost to the UK healthcare system for each individual with an ADHD diagnosis is approximately £860 p.a. (approx. $1430 US). This is an interesting and welcome analysis, not least because it uses the individual as the unit and therefore includes all the resource costs of the associated behaviours such as self-harm and co-morbid conditions such as autism which frequently accompany the presentation of ADHD. Previous cost analyses have estimated an overall cost of ADHD to various national economies [2,3], and such estimates are based on measured prevalence of ADHD. As Holden and colleagues rightly point out, estimates of the prevalence of diagnosed ADHD vary widely. The Holden study used stringent criteria to detect new diagnoses of ADHD from the UK Clinical Practice Database between 1998 and 2008 to provide health service relevant incidence and prevalence figures, and a comparison with age and gender matched controls from the same database to estimate health services resource use. There are many methodological issues that complicate the estimation of prevalence and incidence of diagnosed ADHD. In this article, we discuss differences in prevalence estimates of diagnosed ADHD and the reasons for this.

## Estimating prevalence of ADHD

Holden et al. report the UK prevalence of diagnosed ADHD at 0.5% in 2009 for registered patients aged 6 to 17 years, which is a surprisingly low estimate in comparison with the prevalence of 9.5% for parent-reported diagnoses of ADHD among children aged 4-17 years from USA’s Center for Disease Control (CDC) in 2007 [4], and our own estimate of 1.4% using the same measure of parent-reported diagnosis in the UK among children age 7 also in 2007 [5]. Comparable figures have been derived for diagnosed ADHD in Europe: for example, in Denmark, the prevalence estimate of diagnosed ADHD, calculated from combining records from psychiatric registers in secondary care and methylphenidate use, is that 1.4% of children have an ADHD diagnosis [6].

Diagnoses, of course, particularly child, psychiatric diagnoses, are subject to the vagaries of fashion. Indeed, ADHD has been described as the ‘diagnosis du jour’ by some scholars [7]. A more valuable prevalence estimate is that based on the prevalence of children suffering from symptoms of ADHD at clinical levels in the population. Such estimates are made by epidemiological studies using validated ADHD rating scales, such as the Connors Scale, or standardised diagnostic measures such as the Development and Well Being Assessment (DAWBA). Using the DAWBA, the actual prevalence of children with symptoms of ADHD in the UK population (as opposed to children with ADHD diagnosis), was estimated at 1.5% in 2004 [8].

Even among rigorous epidemiological studies, differences in the samples selected and measures used can make comparisons between figures all but meaningless. Polanski and colleagues, for example, reported prevalence estimates that varied from 1.5% to 25%, from studies across the world [9]. These differences are due, at least in part, to wide differences in the way ADHD is rated and by whom. Additionally, different prevalence estimates in *diagnosed* ADHD may reflect differences in recognition rather than true differences in levels of underlying impairment. DSM criteria, most often used in the US, are less stringent than the ICD-10 criteria that are more often applied in Europe, which may go some way toward explaining higher rates of ADHD reported in the USA [10].

There are on going debates about whether the prevalence of ADHD really is lower in the UK than in the US [11]. Holden et al.’s findings do suggest that ADHD diagnosis is less often used by doctors in the UK than in the USA, but this question of recognition must be separated from estimates of the number of children suffering from these impairing symptoms in the population who may not have been brought to the attention of health services. The identification of ADHD has be shown to vary across geographical region [12], and by ethnicity [13] and gender: girls are less often recognised than boys, as Holden and colleagues point out. Such differences, either cultural, in terms of differences in diagnostic criteria, or arising from the ‘subjectivity of clinicians’ to which the article refers, render the question of whether ADHD is under or over-diagnosed a red herring: the answer depends on where you fix the cut-point for clinical ADHD, and this itself is a moving target [14].

## Conclusion

Perhaps the more pertinent question is not ‘what is the prevalence of diagnosed ADHD’, but whether it is helpful for children to be diagnosed, or for families to have their child diagnosed with ADHD / receive treatment. Making this call involves weighing up the costs versus the benefits of diagnosis of ADHD for each individual child and family. Certainly, for children who are severely impaired, numerous studies show that a range of outcomes at adolescence and adulthood are negatively affected. These include lower academic attainment, fewer employment prospects, and less chance of forming stable long term relationships, as well as increased odds of ending up with a criminal record [15-17]. There is good evidence that treatment with methylphenidate and other anti-ADHD drugs is effective in improving some of these outcomes [18] and can also improve family functioning [19]. There is also evidence to suggest non-pharmacological interventions for childhood ADHD are moderately effective. The extra costs for healthcare services in the UK of $1430 per child estimated by Holden and colleagues may seem high, but given the evidence, this may be inexpensive compared with the long-term costs, both social and economic, of not treating severely affected children.

## Competing interests

The authors have no conflict of interests.

## Authors’ contributions

Both authors have been involved in drafting the manuscript or revising it critically for important intellectual content and have given final approval of the version to be published.
